# The Human Digestive Tract Is Capable of Degrading Gluten from Birth

**DOI:** 10.3390/ijms21207696

**Published:** 2020-10-18

**Authors:** Silvia Fernández-Pérez, Jenifer Pérez-Andrés, Sergio Gutiérrez, Nicolás Navasa, Honorina Martínez-Blanco, Miguel Ángel Ferrero, Santiago Vivas, Luis Vaquero, Cristina Iglesias, Javier Casqueiro, Leandro B. Rodríguez-Aparicio

**Affiliations:** 1 Área de Bioquímica y Biología Molecular, Departamento de Biología Molecular, Facultad de Veterinaria, Universidad de León, 24071 León, Spain; sferp@unileon.es (S.F.-P.); sgutg@unileon.es (S.G.); nnavm@unileon.es (N.N.); hmarb@unileon.es (H.M.-B.); ma.ferrero@unileon.es (M.Á.F.); 2 Área de Microbiología, Departamento de Biología Molecular, Facultad de Ciencias Biológicas y Ambientales, Universidad de León, 24071 León, Spain; jen.perezandres@gmail.com (J.P.-A.); javier.casqueiro@unileon.es (J.C.); 3 Instituto de Biología Molecular, Genómica y Proteómica (INBIOMIC), Universidad de León, 24071 León, Spain; 4 Servicio de Gastroenterología, Hospital Universitario de León, 24008 Léon, Spain; svivasa@gmail.com (S.V.); lvaqa@unileon.es (L.V.); cristina.iglesias.3@gmail.com (C.I.); 5 Instituto de Biomedicina (IBIOMED), Universidad de León, 24071 León, Spain; 6 Servicio de Pediatría, Hospital Universitario de León, 24008 Léon, Spain

**Keywords:** gluten, celiac disease, meconium, gliadinase activity, gliadin peptides, gastrointestinal tract

## Abstract

The human gastrointestinal system has the capacity to metabolize dietary gluten. The capacity to degrade gliadin-derived peptide is present in humans from birth and increases during the first stages of life (up to 6–12 months of age). Fecal samples from 151 new-born and adult non-celiac disease (NCD) volunteers were collected, and glutenase and glianidase activities were evaluated. The capacity of total fecal proteins to metabolize 33-mer, 19-mer, and 13-mer gliadin peptides was also evaluated by high-performance liquid chromatography (HPLC). Feces from new-borns (meconium) showed glutenase and gliadinase activities, and peptidase activity against all three gliadin peptides. Maximal gluten degradative activity was observed in fecal samples from the youngest volunteers (0–12 months old). After the age of nine months, the gluten digestive capacity of gastrointestinal tract decreases and, from ±8 years old, individuals lose the ability to completely degrade toxic peptides. The gastrointestinal proteases involved in gluten digestion: elastase 2A, elastase 3B, and carboxipeptidase A1 are present from earlier stages of life. The human digestive tract contains the proteins capable of metabolizing gluten from birth, even before starting gluten intake. Humans are born with the ability to digest gluten and to completely degrade the potentially toxic gliadin-derived peptides (33-, 19-, and 13-mer).

## 1. Introduction

Gluten is considered to be the mass that remains when wheat dough is washed to remove starch granules and water-soluble constituents [1]. Gluten includes proteins [1] (mainly gliadins and glutenins), but also lipids (up to 10%) and some starches are also present [2].

A normal Western diet includes 13–15 g of gluten/day [3]. During digestion the proteins, partially digested by the action of pepsin in the stomach, pass to the small intestine and are attacked by the pancreatic proteases chymotrypsin, elastase, and carboxypeptidase. Pancreatic enzymes are firstly secreted as inactive precursors and are activated by the action of trypsin secreted by intestinal epithelial cells. Activated pancreatic enzymes convert proteins into free amino acids, which are easily absorbed by the cells of the intestinal wall [4]. However, gluten proteins from wheat, barley, and rye cannot be completely hydrolyzed by human proteases due to the high proline content. Consequently, human gluten digestion generates large peptides (with 10 to >30 amino acid residues) that in celiac disease (CD) patients cross the small intestinal barrier and are deaminated by transglutaminase 2 [5]. In genetically-predisposed individuals expressing the human-leukocyte antigen (HLA)-DQ2.5 or DQ8, some of these peptides (such as the immunodominant 33-mer) trigger the inflammatory cascade of the autoimmune enteropathy known as celiac disease [6].

Epidemiological studies show that CD is a common disorder with a 1–3% prevalence in the general Western population, and it might be therefore considered as a public health problem in European countries and the USA [7]. CD can occur at any age. Indeed, prospective screening studies demonstrate that the disease appears at a very young age (<3 years old) in children with a first-degree relative with CD, and 50% of children with CD and a family history of the disease develop the disease by the age of 3 [8]. However, these studies also reveal significant differences between childhood and adulthood in terms of disease incidence or clinical manifestations. Thus, higher CD incidence is found in childhood compared to adulthood and depending on the study area, incidence may be up to five times higher in childhood [8]. On the other hand, the clinical presentation of CD is partially age-dependent. A tendency towards lower clinical manifestations can be observed as age increases, and symptoms in childhood appear to be more drastic than in adults. For example, the classic clinical malabsorption pattern is frequently observed in children during diagnosis, while classical symptoms occur in less than 25% of adult cases [9]. In general, very young children (<3 years) present severe symptoms, most commonly with chronic diarrhea, abdominal distension, and growth retardation. In contrast, older children and adolescents (≤18 years) present with milder gastrointestinal symptoms which often consist of recurrent abdominal pain, vomiting, or constipation [7,8,10]. In fact, constipation may be the only manifestation in a celiac adult.

Previous results have revealed that adult human feces have a glutenase-deciphered activity in both healthy individuals [1] and CD adult patients [11]. Our group have deciphered some of the proteins responsible for fecal glutenase activity: elastase 3B, elastase 2A, and carboxypeptidase A1. These three gastrointestinal proteases are present in both healthy and celiac individuals and are involved in the human metabolism of gluten [12]. These proteins are able to fully (13- and 19-mer) or partially (33-mer) hydrolyze gliadin peptides that trigger immune-mediated enteropathy in CD patients.

The objective of this work was to evaluate the gluten metabolism capacity from birth to adult age.

## 2. Results

### 2.1. The Capacity to Metabolize Gluten Is Present in Humans from Birth

**Figure 1:**
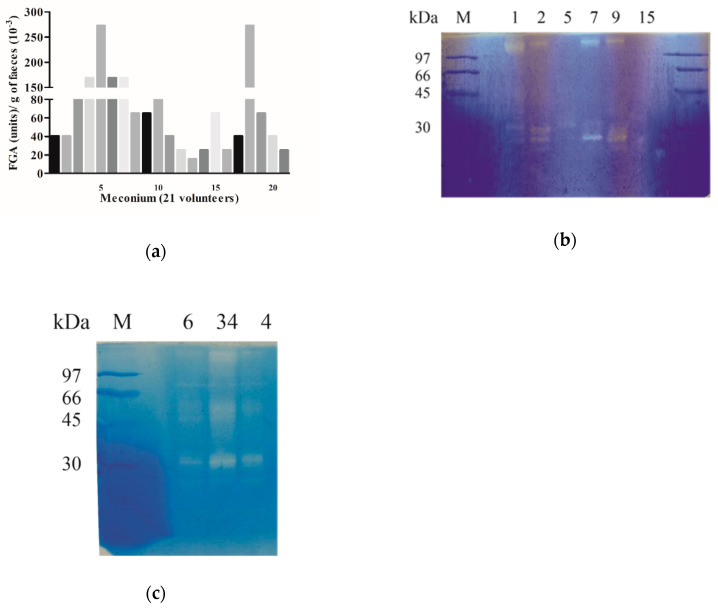
Gluten metabolism in children. (**a**) Bioassay results indicating glutenase activity. Data represent the fecal glutenase activity (FGA) in terms of the number of units of trypsin/g feces of the 21 meconium volunteers. (**b**,**c**) Gliadinase activity. Gliadin zymogram of meconium results from whole-protein fecal samples from (**b**) six newborn volunteers (1, 2, 5, 7, 9, and 15) and (**c**) from three volunteers (4, 6, and 34) two months old. M: electrophoretic molecular weight marker.

**Figure 2:**
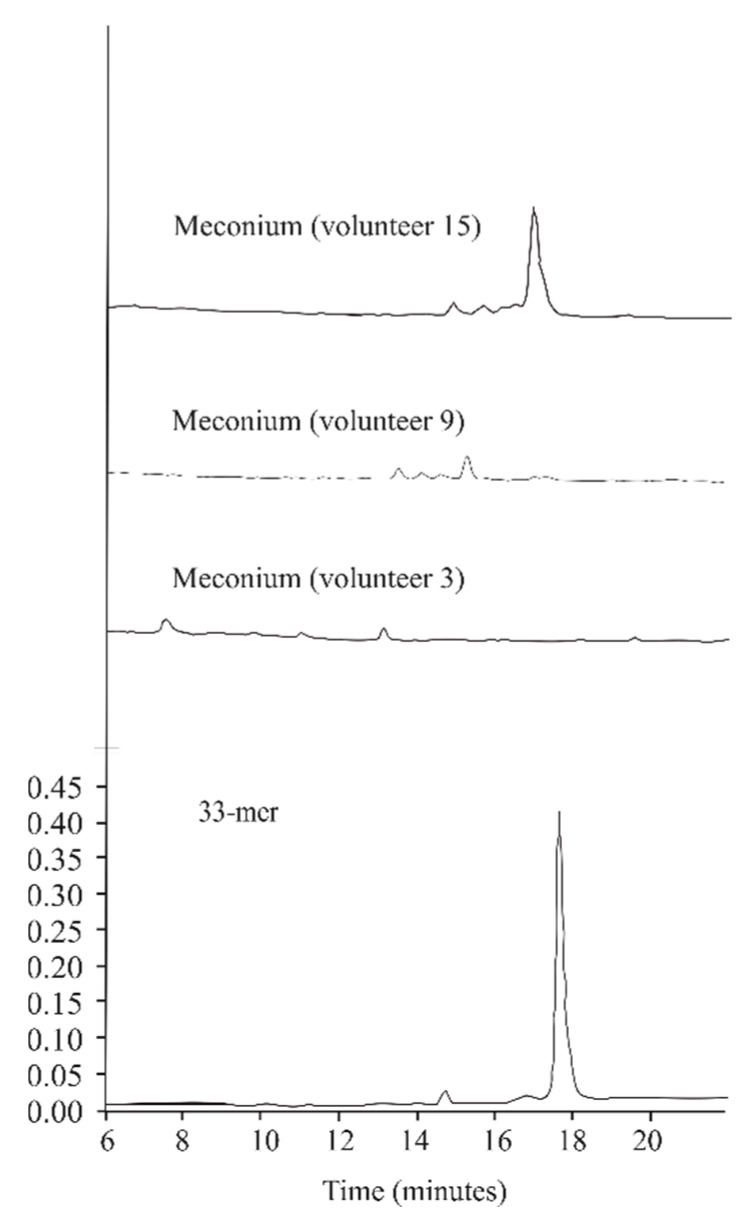
Meconium hydrolytic activity against 33-mer gliadin peptide. High-performance liquid chromatography (HPLC) chromatograms generated after incubating the 33-mer peptide for 60 min at 37 °C with desalted meconium protein extracts from newborn volunteers (3, 9, and 15) chosen as representative hydrolytic models. The bottom of the figure shows the chromatographic migration of the peptide.

To investigate the capacity of the human GI tract to metabolize gluten from the first stages of life, we collected the earliest stool (meconium) from 21 newborn children. We observed that glutenase, gliadinase, and peptidase activity is present from birth (Figure 1 and Figure 2). Fecal glutenase activity (FGA) was confirmed by bioassay (Figure 1a) in all 21 meconium samples and values observed ranged between 15,000 and 272,000 FGA units/g of feces. We also detected gliadinase activity measured by zymography in all meconium samples, using gliadin as substrate, (Figure 1b). Despite the high diversity in total fecal proteins observed by SDS-PAGE, densitometric analysis of zymograms revealed the presence of two principal areas with gliadinase activity corresponding to >97 and 35–25 kDa zones (Figure 1b). In samples from two-month-old patients, a third area of gliadinase activity was detected in the 35–45 kDa range (Figure 1c). The use of HPLC also revealed that the all meconium samples showed hydrolytic activity against the immunogenic 33-mer gliadin-derived peptide.

Figure 2 shows the degradative activity of three representative meconium samples (volunteers 3, 9, and 15). The samples corresponding to volunteers 3 and 9 were able to completely degrade the 33mer peptide under the conditions tested, whereas the sample of volunteer 15 only partially degraded it, therefore generating intermediate peptides.

### 2.2. The Capacity to Degrade Gluten Changes with Age

**Figure 3:**
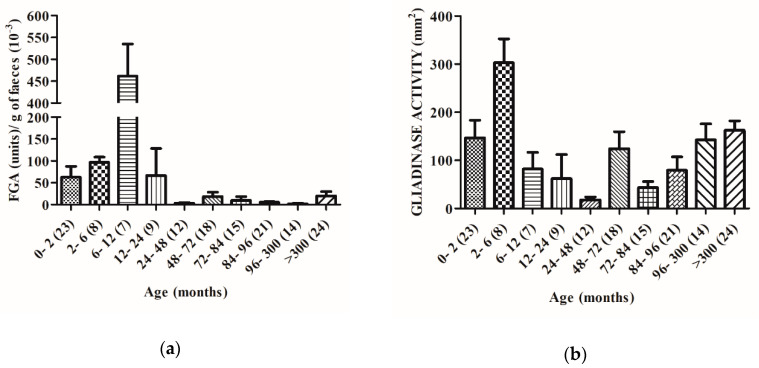
Gluten metabolism in all age range volunteers. (**a**) Glutenase bioassay results of fecal samples from 151 volunteers grouped by age ranges. Data represented as the mean ± SEM in terms of the number of units of trypsin/g feces of each age group. (**b**) Gliadinase activity evaluated by zymography and quantified by densitometry of the hydrolytic areas. Data are represented as the mean ± SEM in terms of the hydrolytic areas in mm^2^. Brackets reflect the number of volunteers of each age group. Interaction of age with glutenase and gliadinase activities is shown by two way ANOVA (*p* = 0.0001).

Feces from healthy non-celiac disease (NCD) volunteers with ages between zero and >300 months were used to evaluate the glutenase activity of the human GI tract. The results obtained were grouped by age ranges (Figure 3). By bioassay, we registered maximal gluten degradative activity (between 37,000 and 535,000 FGA units/g of feces) in the fecal samples from the youngest volunteers (from 0 to 12 months). The highest FGA activity was observed at 6–12 months (Figure 3a). After 12 months (a significant decrease was registered: 12–24 (85%), 24–48 (99%), 48–72 (95%), 72–84 (97%), 84–96 (98%), 96–300 (99%), and >300 months (95%).

These samples were also used to value the specific gliadinase activity. By zymography we observed that in general gliadinase activity (evaluated by densitometric quantification of all the hydrolytic zones generated by each sample), was also significantly higher in the feces from newborns, reaching values between 41 and 341 mm^2^ and in the 2–6 month-old group between 147 and 508 mm^2^. The differences were statistically significant between these groups (Figure 3b). In contrast to FGA, we observed a significant decrease of gliadinase activity in patients older than 6 months (considered 100%): 6–12 (73%) 12–24 (80%), 24–48 (94%), 48–72 (41%), 72–84 (86%), 84–96 (74%), 96–300 (53%), and >300 months (46%).

**Figure 4:**
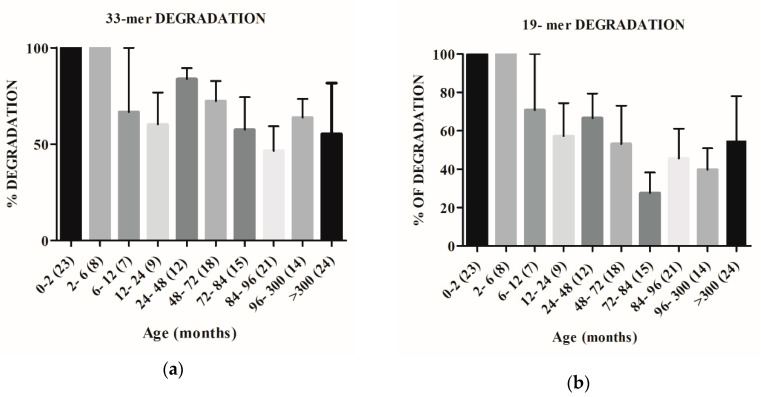

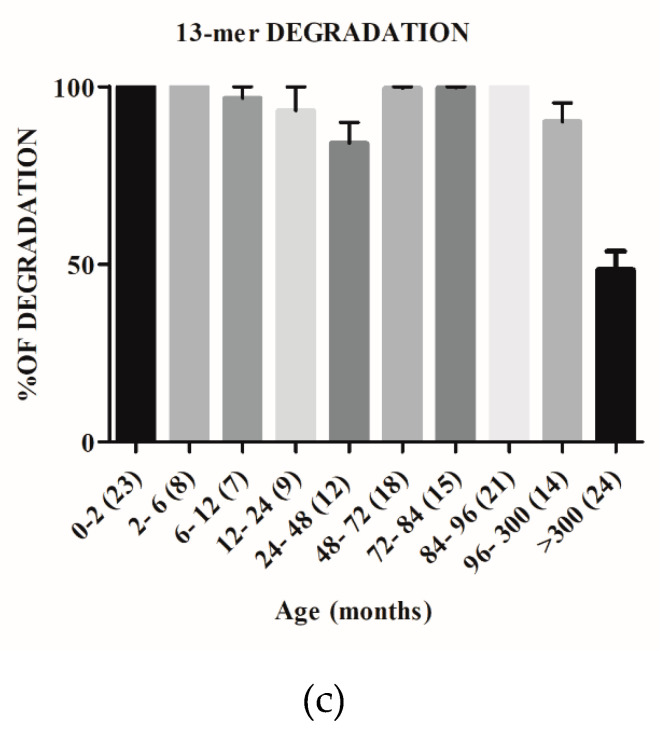
Fecal hydrolytic activity against gliadin peptides derived from samples from different age range volunteers. HPLC chromatograms generated after incubating the 33-, 19-, and 13-mer peptides for 60 min at 37°C with desalted fecal protein extracts were used to evaluate the peptide degradative capacity (in%). The results are grouped by age ranges of samples. Brackets reflect the number of volunteers of each age group. Interaction of age with degradation of immunogenic gluten-derived peptides is shown by two way ANOVA. (**a**) *p* = 0.2128; (**b**) *p* = 0.0905; (**c**) *p* = 0.0001.

We also investigated the capacity of human GI proteases to target immunogenic gliadin-derived peptides by performing “in vitro” digestions of 33-, 19-, and 13-mer peptides by using the extracted proteins from all fecal samples. Figure 4a, confirms the capacity of the younger groups (0–2 and 2–6 months-old) to completely degrade the original 33-mer and shows a downward trend of hydrolytic activity from the 6 month-old infants (6–12 month-olds group) to adult volunteers (>300 months). However, fecal samples of volunteers over 48-months old were unable to completely degrade the original 33-mer, even after 1h of incubation. Similar to 33-mer, samples from new-born volunteers (0–2 month group) showed the ability to completely hydrolyze the 19-mer gliadin derived peptide (Figure 4b). In addition, a downward trend of hydrolytic activity was observed from the youngest (2–6 month group) to adult volunteers (>300 months) and significant statistical differences were registered, in this case, from 6-month-old infants (Figure 4b). Fecal samples from individuals over 6 months old were unable to completely degrade the 19-mer gliadin peptide. Concerning 13-mer, we observed that only the fecal samples from >300 month-old volunteers were not able to complete degrade this gliadin-derived peptide (Figure 4c).

### 2.3. The Gastrointestinal Proteases Involved in Gluten Human Digestion Are Present from Birth

**Figure 5:**
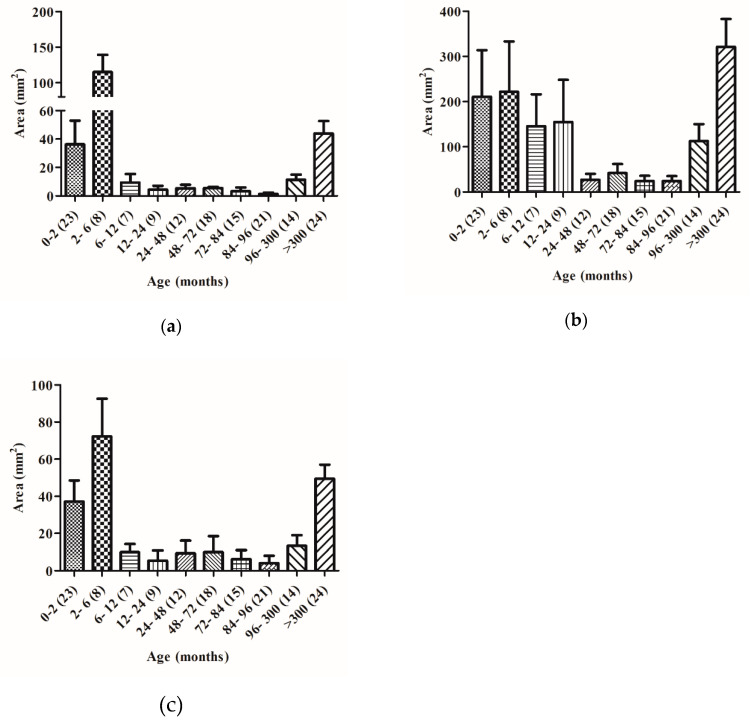
Gliadinase activity of fecal expected carboxypeptidase A1 (CBPA1), elastase 2A (CEL2A), and elastase 3B (CEL3B) from samples from different age range volunteers. Degradative gliadin activity was evaluated by zymography and quantified by densitometry of the corresponding hydrolytic areas: >97 kDa for expected CBPA1 (a), 45–35 kDa for expected CEL2A (b), and 30–25 kDa for expected CEL3B (c). The results are grouped by age ranges of samples. Brackets reflect the number of volunteers of each age group. Interaction of age with gliadinase activity is shown by two way ANOVA. (**a**) *p* = 0.2128; (**b**) *p* = 0.0905; (**c**) *p* = 0.0001.

Previous work, developed in our laboratory, revealed that GI carboxypeptidase A1, elastase 2A, and elastase 3B are involved in gluten digestion in both healthy and CD adult individuals [12]. Zymography studies of meconium samples show two principal zones with gliadinasic activity (Figure 1b) whose molecular weights match with gastrointestinal carboxipeptidase A (>97 kDa), and elastase 3B (30–25 kDa) previously characterized in our laboratory [12]. Gliadinase activity corresponding to the elastase 2A (45–35 kDa) zone was detected in individuals older than 2 months (Figure 1c). By zymography and with fecal samples from 151 healthy non-celiac disease (NCD) volunteers with ages between zero and >300 months using densitometry we evaluated the relative gliadinase activity of these three zones. Figure 5 shows the area of gliadinase activity in the range of carboxipeptidase A (>97 kDa), elastase 2A (45–35 kDa), and elastase 3B (30–25 kDa) zones. The three enzymatic activities were significantly higher in feces from younger individuals: between 0 to 6 month-olds for expected carboxipeptidase A and elastase 3B (Figure 5a,c) and between 2 to 24 month-olds for expected elastase 2A (Figure 5b). After that, a significant decrease was registered (between 81 and 99%) that remained for up to 96 month-old individuals. Finally, a significant increase of the enzymatic activity of the three protease activities was registered (in the range of 96–300 months), and volunteers of >25 years old reached activities levels similar to new-born individuals (Figure 5).

## 3. Discussion

The results presented in this work reveal, for the first time, that feces from new-born human (meconium) contain proteins capable of gluten degradation (Figure 1), which can hydrolyze the potential toxic and immunologic peptides 33-, 19-, and 13-mer gliadin-derived peptides (Figure 2 and Figure 4). Results from zymograms show a gliadinase profile in meconium (Figure 1b) and individuals older than two months (Figure 1c) similar to previously observed in fecal samples from CD and NCD patients [12], suggesting that gluten and gliadin fecal degradation in human newborns is caused by the same proteases that we previously purified and identified using feces from adult NCD individuals [12], namely: elastase 3B (30–25 kDa), elastase 2A (43–35 kDa), and carboxypeptidase A1 (>97 kDa). Thus, our results allow us to conclude that the human digestive tract has the necessary tools for gluten-protein hydrolysis from birth, before starting gluten intake. 

The evaluation of hydrolytic activity reveals the total degradation capacity of new-born fecal samples against gliadin peptides 33-, 19-, and 13-mer (Figure 4), although in some cases, the genesis of intermediate peptides is observed (Figure 2). However, previous results [12] have revealed that the GI proteases elastase 3B, elastase 2A, and carboxypeptidase A1, identified as responsible for the adult fecal glutenase activity, are not able to detoxify immunogenic gliadin derived peptides, especially the 33-mer. Thus, these results indicate that other proteases may be involved in the gliadin metabolism or gliadin-derived peptide hydrolysis. It is possible that minor proteases of microbial origin can participate in degrading the 33-mer. Previous studies have shown that a specific microbiota is present in human meconium [13]. Moreover, it has been described that maternal commensal microbiota presented to the fetus during the last trimester of pregnancy contribute to the establishment of oral tolerance [14]. In this sense, in our laboratory we have isolated bacteria from meconium (*Lactobacillus rhamnosus* and *Rothia* sp), which show both glutenase and peptidase hydrolysis activities [15]. As Caminero et al. [8] showed before, bacterial proteases are involved in gluten metabolism in the human gut. These bacterial proteases can reduce or increase the activity of gliadin-derived peptides against the immune system. For this, we propose that all proteases present in the digestive tract (human and microbial origin) with glutenase activity participate in the degradation of gluten, preventing the genesis of potentially toxic peptides. Furthermore, we support the hypothesis previously reported [16] that suggested that maternal/infant diet, antibiotic exposure, and surrounding environment may significantly affect the early development of the infant intestinal microbiome and therefore the efficiency of gluten digestion.

Our results indicate that both human gluten degradation (Figure 3) and gliadin-derived peptide hydrolysis capacity (Figure 4) are present from birth and increase during the first stages of life (up to 6–12 months of age). Moreover, for up to 12 month-olds, the feces samples are able to degrade all the gliadin-derived peptides assayed: 33-, 19-, and 13-mer (Figure 4). After the age of 6 months, a significant decrease in the fecal gliadin degradative capacity is registered (Figure 3b). This decrease in the degradative capacity of the gliadin is maintained throughout the higher ages (between 46 and 94% with respect to values at 2–6 months old) and although with significant variations, in any time, the maximum levels of the gliadinase activity are recovered (Figure 3b). A similar profile is observed in the evaluation of feces’ hydrolysis capacity against gliadin-derived peptides, especially with regard to 33- and 19-mer. As shown in Figure 4, fecal samples from humans older than 12 months are not able to fully hydrolyze the 33- and 19-mer gliadin-derived peptides. These results reveal that the capacity to metabolize gluten appears to be age-dependent and suggest that neither gluten nor immunogenic derived peptides are responsible for the enhanced activity, at least during the first months of life. Since the fecal glutenase activity is a consequence of the metabolism of the GI tract, all these results also allow us to conclude that humans are born with the ability to digest gluten and to completely degrade the original immunogenic and toxic gliadin-peptides (33-, 19-, and 13-mer). After the age of six months, the digestive capacity of the NCD human gastrointestinal tract decreases and, from ±12 months old, individuals lose the ability to completely degrade toxic peptides. Moreover, the fact that the GI proteases involved in gluten human digestion: expected carboxypeptidase A1, elastase 2A, and elastase 3B, also reach the maximal levels of activity in fecal samples from infants up to 3–6 months old (Figure 5) and the fact that fecal samples from older volunteers (up to 96 months old) show a significant decrease (up to 95%) in the activity of these three degradative proteases (Figure 5), are in agreement with the above conclusions and confirm the fundamental role that these human digestive proteases have in the human metabolism of gluten.

Furthermore, the similar age-profile observed in the studies of these GI protease activities (Figure 5) and both gliadin degradation (Figure 3) and toxic peptide hydrolysis capacity (Figure 4), suggests that modifications in gluten degradative capacity can be also intimately related with changes in the human diet. At birth, the intestine is still immature [17] and during the first stages of life, healthy infants are fed exclusively with breast milk or formula milk. However, at 6–9 months old, normal European children’s diet includes cereals with gluten and other foods that gradually replace breastfeeding. The fact that fecal gluten metabolism activity decreases in samples of individuals from 6 months old (Figure 3b and Figure 4), supports the hypothesis that a gluten-containing diet may negatively influence the human gluten digestive capacity. It is possible that molecules present in the diet [18] such as alfa-amylase/trypsin inhibitors (ATIs), from wheat and barley cereal, or even natural endogenous protease inhibitors, such as serpins or elafin, particularly present in the gastrointestinal tract [4] can modulate the GI proteases involved in the metabolism of gluten (elastase 3B, elastase 2A, and carboxypeptidase A1) and reduce their ability to completely degrade toxic peptides. In this sense we have observed that feces from CD patients can degrade gliadin more effectively than feces from non-CD patients [12]. Previous results have revealed that the fecal glutenase activity from adult CD patients reaches 8000–108,000 FGA units/g feces [12], values that are in the range of the values obtained in this work (20,000–260,000 FGA/g feces) using meconium samples (Figure 1a) and up to 2–5-fold higher than adult NCD individuals [12]. Thus, it is possible that a gluten-free diet avoids the inhibition of GI glutenase activity.

Moreover, changes in the infant diet (from breast milk to gluten-containing cereals) promote changes in the intestinal microbiome [19] that also can modify the efficiency of gluten degradation. The intestinal microbiota constitutes an important source of proteases in the GI tract that participate in the regulation of proteolytic activities of mammalian proteases during homeostasis. In humans, microbial colonization in the gut begins at birth, and the composition and abundance of microbial taxa undergo profound physiological alterations until approximately the age of 3, when becomes “like-adult microbiota” [20]. Differences in microbiota composition may also contribute to justify the modification that we have observed on gluten catabolism capacity between children and adults.

With respect to developing celiac disease, previous results have revealed that in susceptible individuals, the later introduction of gluten does not seem to influence the overall CD [21] but breastfeeding can delay its appearance [22]. Moreover, ATIs have been shown to be potent activators of innate immune responses in CD patients [23]. For all this, we propose that protease inhibitors such as ATIs and serpins can be involved in initial CD development. Could avoiding the presence of these molecules and/or extending breastfeeding over time or delaying the intake of cereals with gluten maintain the ability to completely degrade gliadin and its potentially toxic peptides? We are currently working to answer these questions.

## 4. Materials and Methods

### 4.1. Fecal Sampling

One hundred and fifty-one subjects were included in this study all of them were healthy with no known food intolerances (range 0–60 years). The subjects were symptom-free volunteers for CD. The study complied with the Declaration of Helsinki guidelines, and all procedures involving human subjects were approved by the local ethics committee of the University Hospital of León (approval number 1626, 6 May 2016). Written informed consent was obtained from all subjects or parents. Fresh stools from subjects were collected and immediately stored at −80 °C. Previously, we showed that glutenase activity in stool samples was not modified by freezing at −80 °C. Fecal samples were homogenized and processed immediately after thawing. 

### 4.2. Extraction and Preparation of Fecal Proteins

All procedures were performed at 4 °C unless otherwise indicated, as described by Gutierrez et al. [12] with modifications:

Step 1: Sample preparation: Fecal samples were homogenized by mechanical stirring (90 min) in 10 mM Tris-HCl, pH 7.5 (1:5 *w*/*v*) and centrifuged (3100× *g*, 30 min). The supernatant was re-centrifuged at 30,000× *g* for 20 min.

Step 2: Ammonium sulphate fractionation: Each supernatant obtained from step 1 was precipitated with ammonium sulphate. The 80% ammonium sulphate precipitate (containing glutenase activity) was collected by centrifugation at 30,000× *g*, 20 min. The pellet was dissolved in 10 mM Tris-HCl (pH 7.5) and ammonium sulphate was eliminated by passing through a Sephadex G-25 (PD-10) column (GE Healthcare Live Sources, San Diego, CA USA) equilibrated with Tris-ClH (pH 5) in accordance with manufacturer’s indications.

### 4.3. Evaluation of Fecal Glutenase Activity

Fecal glutenase activities were measured as previously described [1]. Briefly, fecal samples were spread on agar plates (MCG-1) containing 1.5% gluten, 20 g/L glucose, 0.05 g/L CaCl_2_, 0.07 g/L ZnSO_4_, 0.05 g/L L-cysteine, 0.1% Tween 80, 60 mM phosphate buffer (pH 6.5), and 1.5 g/L agar, and incubated at 37°C for 24 h. The glutenase activity (FGA) was evaluated by measuring the diameter of the halo formed. Trypsin diluted in saline was used for construction of a standard curve. Fecal glutenase activity was expressed as trypsin-activity equivalents/g feces [1].

### 4.4. Gel Electrophoresis

SDS-PAGE (12%) was run as described by Laemmli [24] with modifications described by Helmerhorst and Wei [25]. Protein molecular weights were estimated using standard protein markers (97–14.4 kDa; GE Healthcare, Amersham LMW, Dorset UK). Gels were stained with Coomassie Brilliant Blue R-250.

### 4.5. Detection of Gliadinase Activity by Zymography

Gliadin degradation was assessed as previously described [12] using 12% SDS-PAGE zymogram gels containing wheat gliadin (0.6 mg/mL; Sigma, St. Louis, MO, USA), without β-mercaptoethanol. Protein samples were diluted 1/20 in 10 mM Tris-HCl (pH 7.5) and run in electrophoresis gel at 100 V and 4 °C. Afterwards, renaturation of proteins was performed by washing the gels twice for 30 min in renaturing buffer containing 2% (*v*/*v*) Triton-X-100, 0.1 M NaCl, and 0.05 M Tris-HCl (pH 7.8). Next, gels were maintained in developing buffer at 37 °C overnight and stained for 30 min with 0.1% (*w*/*v*) Coomassie Brilliant Blue R-250 [12].

### 4.6. Densitometric Analyses of the Electrophoretic Results

Densitometry analyses of SDS-PAGE and zymogram gels were performed as previously described [12] using lane-based background subtraction, followed by measuring the peak areas using a densitometer (Bio-Rad GS800) and Quantity One 1-D analysis software (Bio-Rad Laboratories Inc., Hercules, CA, USA). The obtained area values were used for statistical analysis using duplicate gels for each sample. Proteins were represented by comparing their relative motilities with those of molecular weight standards.

### 4.7. Peptidase Activity against 33-Mer, 19-Mer, and 13-Mer

Peptides used in this paper were: 33-mer (LQLQPFPQPQLPYPQPQLPYPQPQLPYPQPQPF), 19-mer (LGQQQPFPPQQPYPQPQPF), and 13-mer (LGQQQPFPPQQPY) synthetized by Bionova Científica, S.L. (Madrid, Spain) with purification grade around 96%.

We assayed 33-mer, 19-mer, and 13-mer hydrolysis as described previously [26], with modifications [12]. Briefly, reaction mixtures containing 0.8 mg/mL of desalted protein extract (see step 2.2), 33-, 19-, or 13-mer peptide (60 μM) were incubated at 37 °C for 60 min and then stopped by boiling at 100 °C. 10-µL of each filtered aliquot was subjected to reverse-phase HPLC. The elution phases consisted of (A) MilliQ H_2_O containing 0.1% trifluoroacetic acid (TFA) (*v*/*v*) and (B) acetonitrile and 0.1% TFA (*v*/*v*). The eluate was monitored by measuring UV absorbance at 215 nm. Negative controls included runs with proteins denatured by boiling for 15 min, or 33-, 19-, and 13-mer peptides assayed without protein extract.

### 4.8. Statistical Analysis

Statistical analysis was performed with Two Way ANOVA analysis. Values are considered significant when *p* < 0.05. Data are displayed as the mean ± SE.

## Abbreviations

CD	Celiac disease
FGA	Fecal glutenase activity
GI	Gastrointestinal
LPLC	Low-performance liquid chromatography
NCD	Non-celiac disease
